# Effect of network structure on spike train correlations in networks of integrate-and-fire neurons

**DOI:** 10.1186/1471-2202-12-S1-P272

**Published:** 2011-07-18

**Authors:** Volker Pernice, Benjamin Staude, Stefano Cardanobile, Stefan Rotter

**Affiliations:** 1 Bernstein Center Freiburg and Faculty of Biology, Albert-Ludwig-University, 79104 Freiburg, Germany

## 

Balanced networks of excitatory and inhibitory neurons are a popular paradigm to describe the ground state of cortical activity. Although such networks can assume a state of asynchronous and irregular activity with low firing rates and low pairwise correlations, recurrent connectivity inevitably induces correlations between spike trains [1]. To elucidate the influence of network topology on correlations, we have recently employed the framework of linearly interacting point processes [2] as an analytically tractable model for network dynamics [3]. A power series of the connectivity matrix can be used to disentangle the different contributions to pairwise correlations from direct and indirect interactions between neurons.

In the present study we show that this framework can be applied to approximate dynamics of networks of integrate-and-fire neurons, if the reset after each spike is formally described as self-inhibition. The reset then effectively decreases overall correlations. We study ring networks, where we are able to derive analytical expressions for the distance dependence of correlations and fluctuations in population activity. Rates and correlations in simulated networks are predicted accurately, provided that spike train correlations are reasonably small and the linear impulse response of single neurons is known.
